# Complete hybrid genome assembly of *Mannheimia haemolytica* serotype A2 strain D95 isolated from ovine lung

**DOI:** 10.1128/mra.00552-24

**Published:** 2024-10-14

**Authors:** Anna K. Goldkamp, Harish Menghwar, Rohana P. Dassanayake, Fred M. Tatum, Robert E. Briggs, Eduardo Casas

**Affiliations:** 1 Ruminant Diseases and Immunology Research Unit, National Animal Disease Center, United States Department of Agriculture, Agricultural Research Service, Ames, Iowa, USA; 2 ARS Research Participation Program, Oak Ridge Institute for Science and Education (ORISE), Oak Ridge, Tennessee, USA; SUNY College of Environmental Science and Forestry, Syracuse, New York, USA

**Keywords:** genome sequence, *Mannheimia haemolytica*

## Abstract

*Mannheimia haemolytica* is a major bacterial pathogen associated with broncho- and fibrinous pneumonia in ruminants. Here, we report the complete genome sequence of an isolate of serotype A2 *M. haemolytica* (D95) recovered from a pneumonic ovine lung. The D95 genome has a size of 2.7 Mb and contains 2,720 genes.

## ANNOUNCEMENT

*Mannheimia haemolytica* is a Gram-negative pathogen associated with respiratory disease in ruminants. *M. haemolytica* is commensal in the upper respiratory system but can spread and establish infection in the lower respiratory tract when an animal is immunocompromised (1, 2). Serotypes A1 and A6 predominate in the pneumonic bovine lungs, whereas serotype A2 predominates in the pneumonic small ruminant lungs (3–5). The genome sequence of serotype A2 isolate from ovine could further our understanding of virulence factors linked to host specificity.

The D95 isolate was recovered from pneumonic ovine lung in July 1983 and submitted to the Iowa State University diagnostic laboratory as part of a routine case submission. Isolate D95 was plated on BD BBL Trypticase Soy Agar with 5% sheep blood and incubated at 37°C with 7.5% CO_2_ for 24 hours producing growth consistent with *Mannheimia haemolytica*. The isolate was identified as serotype A2 by plate agglutination and stored in Columbia broth with 10% glycerol at −80°C. A colony of isolate D95 was replated, grown for 24 hours, and submitted for sequencing. Genomic DNA was isolated for short and long-read sequencing using the Promega Whole Blood and Qiagen Puregene Kit, respectively. Libraries were prepared with Nextera XT DNA Library Preparation Kit (Illumina) and sequenced on the MiSeq system (2 × 250 bp), yielding 708,154 reads. The reads were evaluated using FASTQC (version 0.12.1) (6). Adapter sequences were removed using Trimmomatic (version 0.39), and reads were quality filtered (LEADING:3 TRAILING:3 SLIDINGWINDOW:4:20 MINLEN:36), yielding 677,210 reads (7). Size selection for long reads was done using the PacBio Short read Eliminator Kit (SRE XS kit PacBio). Libraries were sequenced using a MinION R10.4.1 flow cell (FLO-MIN114) and Native Barcoding kit-96 (SQK-NBD114-96) of Oxford Nanopore Technologies (ONT), yielding 147,229 reads. Kits were used according to the manufacturer’s instructions. The read *N*_50_ for ONT sequencing before filtering was 23,381 bp. Base calling was done with Guppy-GPU (version 6.5.7), and adapter sequences were removed using Porechop (version 0.2.4) (8, 9). Long reads were filtered (--min_length 1,000) with Filtlong (version 0.2.1), yielding 124,924 reads (10). Hybrid assembly was performed with Unicycler (version 0.5.0) in conservative mode, using quality-filtered Illumina and ONT reads (11). A single circular chromosome was obtained. Genome assembly statistics were obtained using QUAST (version 5.2.0) (12). The completed D95 genome sequence was 2,707,807 bp with a GC content of 41.12% and a depth of coverage of ~740×. Compared to bovine *M. haemolytica* serotype A2 strain D171 (accession CP006573), the reference coverage was 97.09%. Annotation was accomplished using the NCBI Prokaryotic Genome Annotation Pipeline (2023-10-03.build7061) (13). D95 contained 2,720 genes, including 2,562 protein-coding genes, 88 RNA genes, and 70 pseudogenes. ResFinder (22 March 2024) determined that antimicrobial resistance genes were absent in the D95 genome (14, 15). In D171 and D95 genomes, genes encoding WecB and WecC proteins were missing from the capsule biosynthetic locus, which is a feature of A2 serotypes (16).

## Data Availability

The genome sequence of *M. haemolytica* D95 was deposited in GenBank (CP155540). The fastq files generated are available in the NCBI SRA database (SRR28971289 and SRR28971288).

## References

[1] Clawson ML, Murray RW. 2014. Pathogen variation across time and space: sequencing to characterize Mannheimia haemolytica diversity. Anim Health Res Rev 15:169–171. doi:10.1017/S1466252314000188 25381881

[2] Singh K, Ritchey JW, Confer AW. 2011. Mannheimia haemolytica: bacterial-host interactions in bovine pneumonia. Vet Pathol 48:338–348. doi:10.1177/0300985810377182 20685916

[3] Zecchinon L, Fett T, Desmecht D. 2005. How Mannheimia haemolytica defeats host defence through a kiss of death mechanism. Vet Res 36:133–156. doi:10.1051/vetres:2004065 15720968

[4] Highlander SK. 2001. Molecular genetic analysis of virulence in Mannheimia (pasteurella) haemolytica. Front Biosci:D1128–50. doi:10.2741/highland 11532607

[5] Klima CL, Alexander TW, Hendrick S, McAllister TA. 2014. Characterization of Mannheimia haemolytica isolated from feedlot cattle that were healthy or treated for bovine respiratory disease. Can J Vet Res 78:38–45. 24396179 PMC3878007

[6] Andrews S. 2010. FastQC: a quality control tool for high throughput sequence data. Available from: http://www.bioinformatics.babraham.ac.uk/projects/fastqc/

[7] Bolger AM, Lohse M, Usadel B. 2014. Trimmomatic: a flexible trimmer for Illumina sequence data. Bioinformatics 30:2114–2120. doi:10.1093/bioinformatics/btu170 24695404 PMC4103590

[8] Wick R. 2018 Porechop: adapter trimmer for Oxford Nanopore reads. https://github.com/rrwick/Porechop.

[9] Sherathiya VN, Schaid MD, Seiler JL, Lopez GC, Lerner TN. 2021. GuPPy, a Python toolbox for the analysis of fiber photometry data. Sci Rep 11:24212. doi:10.1038/s41598-021-03626-9 34930955 PMC8688475

[10] Wick R. 2021 Filtlong: quality filtering tool for long reads. https://github.com/rrwick/Filtlong.

[11] Wick RR, Judd LM, Gorrie CL, Holt KE. 2017. Unicycler: resolving bacterial genome assemblies from short and long sequencing reads. PLoS Comput Biol 13:e1005595. doi:10.1371/journal.pcbi.1005595 28594827 PMC5481147

[12] Gurevich A, Saveliev V, Vyahhi N, Tesler G. 2013. QUAST: quality assessment tool for genome assemblies. Bioinformatics 29:1072–1075. doi:10.1093/bioinformatics/btt086 23422339 PMC3624806

[13] Tatusova T, DiCuccio M, Badretdin A, Chetvernin V, Nawrocki EP, Zaslavsky L, Lomsadze A, Pruitt KD, Borodovsky M, Ostell J. 2016. NCBI prokaryotic genome annotation pipeline. Nucleic Acids Res 44:6614–6624. doi:10.1093/nar/gkw569 27342282 PMC5001611

[14] Camacho C, Coulouris G, Avagyan V, Ma N, Papadopoulos J, Bealer K, Madden TL. 2009. BLAST+: architecture and applications. BMC Bioinformatics 10:421. doi:10.1186/1471-2105-10-421 20003500 PMC2803857

[15] Bortolaia V, Kaas RS, Ruppe E, Roberts MC, Schwarz S, Cattoir V, Philippon A, Allesoe RL, Rebelo AR, Florensa AF, et al.. 2020. ResFinder 4.0 for predictions of phenotypes from genotypes. J Antimicrob Chemother 75:3491–3500. doi:10.1093/jac/dkaa345 32780112 PMC7662176

[16] Lo RYC, McKerral LJ, Hills TL, Kostrzynska M. 2001. Analysis of the capsule biosynthetic locus of Mannheimia (Pasteurella) haemolytica A1 and proposal of a nomenclature system. Infect Immun 69:4458–4464. doi:10.1128/IAI.69.7.4458-4464.2001 11401986 PMC98519

